# Measurement invariance of the short Warwick-Edinburgh Mental Wellbeing Scale and latent mean differences (SWEMWBS) in young people by current care status

**DOI:** 10.1007/s11136-021-02896-0

**Published:** 2021-05-28

**Authors:** Rebecca Anthony, Graham Moore, Nicholas Page, Gillian Hewitt, Simon Murphy, G. J. Melendez-Torres

**Affiliations:** 1 Centre for Development, Evaluation, Complexity, and Implementation in Public Health Improvement (DECIPHer), School of Social Sciences, Cardiff University, 1-3 Museum Place, Cardiff, CF10 3BD UK; 2 Wolfson Centre for Young People’s Mental Health, Cardiff University, Cardiff, UK; 3 College of Medicine and Health, South Cloisters, University of Exeter, St Luke’s Campus, Heavitree Road, Exeter, EX1 2LU UK

**Keywords:** Mental wellbeing, Adolescents, Looked after, Care, Measure, Psychometric properties

## Abstract

**Purpose:**

Studying mental wellbeing requires the use of reliable, valid, and practical assessment tools, such as the Short version of the Warwick-Edinburgh Mental Wellbeing Scale (SWEMWBS). Research on the mental wellbeing of children in care is sparse. The current study aims to: (1) examine the unidimensionality of SWEMWBS; (2) assess measurement invariance of SWEMWBS across children and young people in care compared to their peers not in care; and (3) investigate the latent factor mean differences between care status groups.

**Methods:**

We used data from the 2017 School Health Research Network Student Health and Wellbeing (SHW) survey, completed by 103,971 students in years 7 to 11 from 193 secondary schools in Wales. The final data include a total of 2,795 participants (46% boys), which includes all children in care and a sub-sample of children not in care who completed the SWEMWBS scale fully and answered questions about their living situation.

**Results:**

Confirmatory factor analysis supported the unidimensionality of SWEMWBS. The SWEMWBS is invariant across groups of young people in foster, residential and kinship care compared to children and young people not in care at configural, metric and scalar levels. Findings from latent mean comparisons showed that young people in care reported lower mental wellbeing than their peers, with those in residential care reporting the lowest scores.

**Conclusions:**

Findings suggest that SWEMWBS is a valid scale for measuring differences in mental wellbeing for young people in care similar to the population.

**Supplementary Information:**

The online version contains supplementary material available at 10.1007/s11136-021-02896-0.

## Background

Mental wellbeing has emerged as an important construct in population health, described as a fundamental human right and essential for a sustainable and functional society [1]. Mental wellbeing has been defined as ‘a state of wellbeing in which every individual realises his or her own potential, can cope with the normal stresses of life, can work productively and fruitfully, and is able to make a contribution to her or his community’ [2], encompassing concepts such as resilience, self-efficacy and optimism [3]. As opposed to mental illness, which is either prevented or treated, mental wellbeing can only be promoted [4], and promotion has been shown to relate to improved health and longevity in adults [5]. Despite this, little information is available on the prevalence or social patterning of mental wellbeing in young people [3], particularly compared to the extensive data on mental illness [6].

In the United Kingdom (UK), local authority care includes the provision of accommodation for children and young people who are unable to live with their parents. There are a variety of reasons for young people to enter care, with approximately two thirds entering care due to abuse and neglect [7]. As of March 2019, 6846 young people in Wales were in the care of their local authority, the majority (71%) accommodated in foster care placements [7]. While most young people in care in the UK report their experiences to be good [8], and report satisfaction with their life [9], there is clear evidence that those who have experienced care do not fare as well as the general population in relation to their physical health, cognitive and language skills [10], and mental health [11–13], which in turn can impact their development and journey to adulthood [14–16]. Studies have begun to investigate subjective wellbeing of children and young people in foster care in the UK [9], and foster and residential care internationally [17–19]. These studies have consistently identified lower levels of subjective wellbeing of those in care compared to their peers not in care, with those in residential care demonstrating the lowest levels of wellbeing.

Studying mental wellbeing requires the use of reliable measurement tools. The Warwick–Edinburgh Mental Wellbeing Scale (WEMWBS) was developed in 2007 [20] and is one of the most widely used measures of mental wellbeing [21]. It contains 14-items covering both psychological functioning and subjective wellbeing facets of mental wellbeing. A brief seven-item version (SWEMWBS) was subsequently developed using the Rasch measurement model which had preferable psychometric properties to the full version, though it is focussed more on functioning than subjective aspects of mental wellbeing [22]. While measures of subjective wellbeing have been developed for young people in foster care, including specific care-related aspects such as birth parent contact [23], a brief measure of mental wellbeing may be of particular use in population research where practical constraints often restrict the scope for detailed surveys.

It is often assumed that scores represent the same level of the construct for members of different groups. However, the nature and magnitude of relationships between items and a latent construct may differ across groups, meaning that comparisons between groups cannot be meaningfully made unless the measure is capturing the same thing in each sub-group [24–26]. Thus, if we want to know if policies and interventions are working as well for children in care as for the rest of the population, we need to be able to measure this equally well in both groups. Testing for invariance of measures makes it possible to verify whether the members of different groups or cultures ascribe the same meanings to the items of a questionnaire [27], which is critical for informing both practice and research [28]. Most studies examining the psychometric properties of SWEMWBS have been undertaken in adults [22, 29, 30]; the few studies conducted with adolescents have found acceptable measurement invariance properties by age and gender [31, 32], and demonstrate good external construct validity [32].

### The present study

The aims of the present study were to: (1) Confirm the unidimensionality of SWEMWBS; (2) assess measure invariance of SWEMWBS across children and young people in care compared to their peers not in care; and (3) undertake comparison of mean differences in mental wellbeing across those groups. While other specific mental health measures, such as the Strengths and Difficulties Questionnaire [33], have been examined across groups of care-experienced children and young people, to the best of our knowledge no study has yet examined the equivalence of SWEMWBS across these groups.

## Method

We used a population sample (*N* = 2795) of young people in Wales to examine the measurement invariance properties of SWEMWBS across groups of young people currently in the care of the local authority (i.e. foster, residential or kinship care placements) compared to their peers of a similar age not in care.

### Study sample

We used data from the 2017 School Health Research Network Student Health and Wellbeing (SHW) survey, reported in detail elsewhere [32, 34, 35]. The survey was completed by *N* = 103,971 students in years 7 to 11, representing 193 secondary schools in Wales. The SHW survey is an online, closed response, self-completion survey, available in English and Welsh. The survey measures self-reported health behaviours among school students aged 11–16 years (i.e. years 7 to 11 of the British secondary school system), and includes questions from the current round of the international HBSC survey [36] alongside additional questions reflecting current policy, practice and research priorities in Wales. Students completed the survey during school hours between September and December of the autumn term of the 2017–2018 school year and could opt out of the survey. A total of *n* = 79,297 completed the scale fully and answered questions about their living situation. At the time of the survey, *n* = 77,588 (97.84%) were not currently living in care*, n* = 513 (0.65%) currently lived-in local authority foster care, *n* = 126 (0.16%) currently lived-in local authority residential care and *n* = 1,070 (1.33%) were in kinship care. Due to simulation studies showing that severely unbalanced group size conditions are more likely to reduce power and can mask violations of invariance [37, 38], rather than include all children not living in local authority care a sub-sample of children and young people not in care were randomly selected using the ‘sample’ command in Stata to be similar in size to the largest care group (approximately 1.4% of the cases) (*n* = 1086). Thus, the final sample included *N* = 2,795 children and young people.

### Measures

#### Care status

All respondents were asked the following question to assess their current living arrangements. “All families are different (for example, not everyone lives with both their parents; sometimes people live with just one parent, they have two homes, or live with two families) and we would like to know about yours. Please answer this question for the home where you live all or most of the time and tick the ADULTS who live there”. The options included mother, father, mothers partner, fathers partner, grandparent(s), aunt(s)/uncle(s), adult brother(s)/sister(s), foster parents, residential care or a children’s home, independently (on my own or with friends or my partner), and someone or somewhere else. Responses were then categorised into ‘not in care’, ‘foster care’, ‘residential care’ or ‘kinship care’. Observations where students either responded ‘I do not want to answer’, left a question blank or answered yes to eight or more possible living arrangement options (considered implausible) were set to missing.

#### SWEMWBS

All students were presented with the seven questions comprising SWEMWBS [20]: ‘I’ve been feeling optimistic about the future’, ‘I’ve been feeling useful’, ‘I’ve been feeling relaxed’, ‘I’ve been dealing with problems well’, ‘I’ve been thinking clearly’, ‘I’ve been feeling close to other people’, and ‘I’ve been able to make up my own mind about things’ alongside the following question: ‘Below are some statements about feelings and thoughts. Please select the option that best describes your experience of each over the last 2 weeks’. For each question, students could select one of five frequency options: ‘none of the time’, ‘rarely’, ‘some of the time’, ‘often’ and ‘all of the time’.

### Statistical analysis

Stata v.14 [39] was used to conduct descriptive statistics (i.e. minimum, maximum, mean, and standard deviation) for each sample across all seven items of the SWEWMBS scale and to estimate polychoric correlation matrices for the whole sample and by care status.

Following this, lavaan 0.6–3 [40] and semTools 0.5–1 [41] R packages were used to conduct confirmatory factor analysis (CFA) to test the unidimensionality of the SWEMWBS scale and measurement invariance tests. Categorical confirmatory factor analysis was performed using diagonally weighted (DWLS) estimators suitable for ordinally scaled responses [42]. All standardised factor loadings within this single factor should be above 0.5 and statistically significant [43] to support the unidimensionality. Model fit was assessed using Chi-square (*χ*^2^) and its degrees of freedom (test values associated with *p* > 0.05), the Comparative-of-Fit Index (CFI; values ≥ 0.90), Tucker–Lewis Index (TLI; values ≥ 0.90), Root Mean Square Error of Approximation (RMSEA; values close to 0.06) and its 95% confidence interval (CI), and Standardised Root Mean Square Residual (SRMR; values ≤ 0.08) as advised [44].

In accordance with Jöreskog’s strategy [45], we used a multiple-groups structural equation model with successively greater constraints that tested for configural invariance and scalar invariance [46, 47], following the recommended procedure and syntax described by Svetina et al. (2020) [48]. Because of the ordinal nature of the individual items, we again used a diagonally weighted least squares estimator with a scale-shifted test statistic. We first estimated a baseline (configural) where thresholds and loadings are estimated freely using delta parameterization. Next, we estimated a model where the thresholds where constrained to be equal, and finally we estimated a model where both the thresholds and loadings (scalar) are constrained to be equal. The scalar invariance test is a strong invariance test and the establishment of this test is required before the latent means can be compared across groups.

The performance of fit indices for invariance tests with categorical or ordinal data has not been adequately studied [49] and is still a developing area, particularly for ordinal data [50] where there remains a lack of agreement among scholars as to which recommendation to adopt [48]. However, based on recommendations and the size of our sample, we did not use traditional *χ*^2^ tests for invariance. Instead, we used the comparative fit index (CFI) and the root mean squared error of approximation (RMSEA). The CFI has previously been shown to be an appropriate index of measurement invariance, with decrements of greater than − 0.01 in successive models suggesting that measurement variance is not appropriate [51]. In addition, emerging evidence shows promise for the RMSEA as an information criterion, where the lowest value indicates the model with the best trade-off between fit and complexity [52].

Following measurement invariance testing, we compared latent mean differences between care status group. Specifically, a full scalar invariance model was used as the baseline. To compare differences in latent means between groups, we constrained the ‘not in care’ latent mean to 0 and the latent means of the foster care, kinship care and residential care groups were free to estimate [53].

## Results

Descriptive statistics for the sample can be found in Table 1. The response category proportions as well as item means, and standard deviations (data treated as continuous) can be found in Table 2. Across all items, it is worth noting that most children and young people positively rate items assessing their mental wellbeing. Visual examination of polychoric correlation matrices showed significant interitems correlation coefficients, ranging from 0.30 to 0.56 for the full sample (see Online Table S1). The matrices indicated increasing intercorrelation in the different care-experienced groups, with the strongest intercorrelations within the residential care group (ranging from 0.61 to 0.77). The scale demonstrated good internal consistency reliability across all groups (*α* = 0.84 in full sample, *α* = 0.82 ‘not in care’ group; *α* = 0.86 in Foster Care, *α* = 0.90 in Residential Care and *α* = 0.81 ‘kinship care group).

**Table 1:** Sample descriptive statistics (N/%)

Variable	Total sample (*N* = 2795)	Not in care (*N* = 1086)	Foster care (*N* = 513)	Residential care (*N* = 126)	Kinship care (*N* = 1070)
Gender					
Male	1273 (46)	506 (47)	234 (46)	59 (47)	474 (44)
Female	1464 (52)	571 (53)	263 (51)	53 (42)	577 (54)
Prefer not to say	58 (2)	9 (1)	16 (3)	14 (11)	19 (2)
School year					
Year 7	513 (18)	199 (18)	106 (21)	25 (20)	183 (17)
Year 8	550 (20)	213 (20)	103 (20)	25 (20)	209 (20)
Year 9	617 (22)	242 (22)	110 (21)	28 (22)	237 (22)
Year 10	570 (20)	210 (19)	108 (21)	33 (26)	219 (20)
Year 11	545 (20)	222 (20)	86 (17)	15 (12)	222 (21)
Family affluence					
Low	1022 (39)	359 (34)	150 (32)	38 (35)	475 (46)
Medium	822 (31)	325 (31)	143 (30)	36 (33)	318 (31)
High	810 (31)	365 (35)	179 (38)	34 (31)	232 (23)
Ethnicity					
White British	2307 (85)	915 (86)	412 (83)	72 (60)	908 (87)
White non-British	158 (6)	46 (4)	45 (9)	16 (13)	51 (5)
Black and Minority Ethnic	256 (9)	98 (9)	42 (8)	33 (27)	83 (8)
Language					
English	2691 (96)	1029 (95)	499 (97)	117 (93)	1046 (98)
Welsh	104 (4)	57 (5)	14 (3)	9 (7)	24 (2)

**Table 2:** Item descriptive statistics

SWEMWBS items*	‘none of the time’ (N/%)	‘rarely’ (N/%)	‘some of the time’ (N/%)	‘often’ (N/%)	‘all of the time’ (N/%)	Mean ± SD
*Item 1* “I’ve been feeling optimistic about the future”	379 (13.56)	562 (20.11)	830 (29.70)	678 (24.26)	346 (12.38)	3.02 ± 1.22
*Item 2* “I’ve been feeling useful”	320 (11.45)	579 (20.72)	964 (34.49)	667 (23.86)	265 (9.48)	2.99 ± 1.13
*Item 3* “I’ve been feeling relaxed”	249 (8.91)	510 (18.25)	803 (28.73)	806 (28.84)	427 (15.28)	3.23 ± 1.18
*Item 4* “I’ve been dealing with problems well”	323 (11.56)	534 (19.11)	804 (28.77)	751 (26.87)	383 (13.70)	3.12 ± 1.21
*Item 5* “I’ve been thinking clearly”	247 (8.84)	492 (17.60)	832 (29.77)	769 (27.51)	455 (16.28)	3.25 ± 1.18
*Item 6* “I’ve been feeling close to other people”	236 (8.44)	419 (14.99)	667 (23.86)	783 (28.01)	690 (24.69)	3.46 ± 1.24
*Item 7* “I’ve been able to make up my own mind about things”	171 (6.12)	300 (10.73)	582 (20.82)	841 (30.09)	901 (32.24)	3.72 ± 1.20

*WEMWBS is protected by copyright. Those wishing to use WEMWBS can obtain a licence to do so. Please go to https://warwick.ac.uk/wemwbs/using for information on the type of licence you will require and details on how to apply

### Factorial structure

Categorical confirmatory factorial analysis was used to test a unidimensional model in which the scores obtained for the 7 items of the scale all contribute to the evaluation of children and young people’s mental wellbeing. Considering the sensitivity of the chi‐square statistic to sample size [54], we assessed a number of additional indices. Model fit was assessed to be adequate as despite the significant Chi-square all other indices showed excellent fit (*χ*^2^ (df = 14) = 190.75, *p* < 0.001; CFI = 0.988; TLI = 0.982; RMSEA = 0.067 [0.059-0.0.076]; SRMR = 0.024). Furthermore, all standardised factor loadings were statistically significant (*p* < 0.001) and ranged from 0.548 to 0.814 (item 1 = 0.548; item 2 = 0.703; item 3 = 0.701; item 4 = 0.743; item 5 = 0.814; item 6 = 0.657; item 7 = 0.718), higher than the threshold of 0.5 [43]. See Online Table S2 for item factor loadings.

### Measurement invariance

Having verified unidimensionality of the SWEMWBS, we estimated a one-factor configural invariance model. Results from each of the successively stricter invariance tests are reported in Table 3. Configural invariance (baseline model) provided an acceptable fit to the data, (CFI 0.984, TLI 0.958, RMSEA 0.073, *p* < 0.001), meaning the constructs had similar patterns of free and fixed loadings across groups [46]. Metric invariance was subsequently tested whereby factor variances remained freely estimated but factor loadings were held invariant. Findings showed that the model fit the data well and there was no change in CFI (0.000) and a reduction in RMSEA (− 0.017), thus the items therefore loaded onto factors similarly across groups [46]. Scalar invariance was then tested whereby indicator thresholds were now also held invariant, fit indices suggested acceptable fit with this constraint as indicated by little change to CFI (0.003) and a reduction in RMSEA (− 0.010).

**Table 3:** Measurement invariance tests of SWEMWBS across care status

Model constraints	CFI scaled	RMSEA scaled (90% CI)	CFI	RMSEA
Configural	0.984	0.073 (0.068–0.082)	–	–
Loadings	0.984	0.056 (0.049–0.063)	No change	− 0.017
Loadings, thresholds	0.987	0.046 (0.040– 0.053)	0.003	− 0.010
Additive^a^			0.003	− 0.027

^a^Additive change from baseline

### Latent mean differences in mental wellbeing

Based on the establishment of scalar invariance across care status groups, latent mean comparisons can be made between care status groups. The NIC (not in care) group served as the reference group. Findings showed that young people currently in all types of care reported significantly lower mental wellbeing scores than those not in care (kinship care: *E*_st_ = − 0.364 ± 0.051, *p* < 0.001; foster care: *E*_st_ = − 0.319 ± 0.075, *p* < 0.001), and those in residential care reported the lowest levels of mental wellbeing; (*E*_st_ = − 0.882 ± 0.192, *p* < 0.001).

## Discussion

In this study, we analysed the short version of the WEMWBS in a population-based sample of school-aged students to explore measurement invariance and latent mean differences between young people currently in care (foster, kinship and residential settings) and those not currently in the care of local authority. The 1-factor *CFA* test showed that the SWEMWBS exhibited satisfactory model fit and demonstrated unidimensionality, thus this short single-factor instrument may be useful in reducing respondent burden in future studies. The current study established configural, metric and scalar invariances across care status groups, suggesting that differences in SWEMWBS scores between care status groups can be attributed to differences in the underlying latent trait rather than to the measure itself. Researchers employing SWEMWBS in future studies can compare the mental wellbeing scores meaningfully across those in different types of local authority care compared to their peers not in care.

Our findings revealed that young people in all types of care reported significantly lower mental wellbeing scores than their peers of the same age not in care. This result is consistent with findings from previous studies testing wellbeing scores using traditional methods [17–19]. Research shows that developmentally specific factors including parents' availability and wellbeing, family relationships and interactions, quality of care, and supportive learning environments are critical for children’s wellbeing [55]. Thus, given that the majority of children entered care due to abuse or neglect [7] and the strong evidence base showing the long-lasting impact of early trauma and adversity [56–59] we suggest that the lower mental wellbeing of those in care may be due to early negative experiences prior to or during care; however, we do not have the data available to test this assumption. Given that the purpose of the care system is to address these factors by: protecting children from further harm, addressing a child’s need for good parenting, and enabling them to recover from traumatic experiences [9], further work to promote mental wellbeing of young people in care is needed. A scoping review [6] highlighted a number of interventions which may be beneficial in improving children and young people’s wellbeing. The review also highlighted a decreasing emphasis on wellbeing as children grew into teens or young adults, with more interventions available for this age group which intervene in the development of mental illness rather than promoting wellbeing.

Our analysis has several strengths, but also several limitations. Our large-scale nationally representative sample provides evidence of the utility of SWEMWBS for measuring mental wellbeing among young people in care in the UK. A limitation of this study is the lack of testing for invariance across other categories, such as gender, family affluence and ethnicity. Previous studies have shown that SWEMWBS demonstrates strong measurement invariance across sex and age differences in adults [60] and a further study showed measurement invariance across the full age range of secondary school students [32]; however, as previous research has shown that these factors are all connected to strong structural inequities [61], future work should address this limitation by testing for invariance across family affluence and ethnicity. Furthermore, it is possible that results from Wales may not generalise internationally, though evidence of the psychometric properties of SWEMWBS in adults is consistent across multiple cultures [62]. Self-reported data may have been biased by standard limitations (e.g. memory recall biases, social desirability, etc.). While the living situation question enabled us to identify that the ‘kinship care’ group are living with family other than their parents we cannot be sure if they are subject to a formal care order. As the SHW survey is only completed by young people in mainstream schooling, the views of children not in mainstream school are not included, this is particularly significant given that approximately 40% of children in care attend non-mainstream schools such as special schools, pupil referral units and alternative provisions [63].

## Conclusions

This study adds to the growing evidence that SWEMWBS is appropriate for measuring mental wellbeing in young people in care. Additionally, the wellbeing of young people in foster, kinship and residential placements was significantly lower than their peers not in care, highlighting the need for interventions to promote mental wellbeing in this group.

## Supplementary Information

Below is the link to the electronic supplementary material.

Supplementary file1 (DOCX 20 kb)

## Data Availability

(Data transparency). Data underpinning this analysis is available upon reasonable request to the School Health Research Network and completion of the necessary data application documentation.

## Code availability

(software application or custom code) Code is available on request.
