# Supplementary material for: Measurement invariance of the short Warwick-Edinburgh Mental Wellbeing Scale and latent mean differences (SWEMWBS) in young people by current care status
Source: Qual Life Res. 2021 May 28;31(1):205–13. doi: 10.1007/s11136-021-02896-0 (PMC8800901; doi:10.1007/s11136-021-02896-0)
Supplement: Supplementary file 1 — Supplementary file1 (DOCX 20 kb) [file 11136_2021_2896_MOESM1_ESM.docx]

**Measurement Invariance of the Short Warwick-Edinburgh Mental Wellbeing Scale and Latent Mean Differences (SWEMWBS) in Young People by Current Care Status**

Online Supplement

Online Table 1. Overall and care status specific polychoric correlation matrices.

| All groups | | | | | | | |
| --- | --- | --- | --- | --- | --- | --- | --- |
|  | Q1 | Q2 | Q3 | Q4 | Q5 | Q6 | Q7 |
| Q1 | 1.00 |  |  |  |  |  |  |
| Q2 | 0.47 | 1.00 |  |  |  |  |  |
| Q3 | 0.34 | 0.49 | 1.00 |  |  |  |  |
| Q4 | 0.38 | 0.52 | 0.53 | 1.00 |  |  |  |
| Q5 | 0.44 | 0.55 | 0.58 | 0.62 | 1.00 |  |  |
| Q6 | 0.35 | 0.47 | 0.46 | 0.49 | 0.51 | 1.00 |  |
| Q7 | 0.37 | 0.46 | 0.50 | 0.53 | 0.61 | 0.50 | 1.00 |
| *Not in care* | | | | | | | |
|  | Q1 | Q2 | Q3 | Q4 | Q5 | Q6 | Q7 |
| Q1 | 1.00 |  |  |  |  |  |  |
| Q2 | 0.43 | 1.00 |  |  |  |  |  |
| Q3 | 0.30 | 0.45 | 1.00 |  |  |  |  |
| Q4 | 0.38 | 0.45 | 0.47 | 1.00 |  |  |  |
| Q5 | 0.41 | 0.49 | 0.55 | 0.56 | 1.00 |  |  |
| Q6 | 0.30 | 0.41 | 0.43 | 0.42 | 0.46 | 1.00 |  |
| Q7 | 0.31 | 0.39 | 0.47 | 0.47 | 0.56 | 0.45 | 1.00 |
| *Foster care* | | | | | | | |
|  | Q1 | Q2 | Q3 | Q4 | Q5 | Q6 | Q7 |
| Q1 | 1.00 |  |  |  |  |  |  |
| Q2 | 0.53 | 1.00 |  |  |  |  |  |
| Q3 | 0.37 | 0.50 | 1.00 |  |  |  |  |
| Q4 | 0.41 | 0.56 | 0.57 | 1.00 |  |  |  |
| Q5 | 0.45 | 0.58 | 0.64 | 0.65 | 1.00 |  |  |
| Q6 | 0.39 | 0.48 | 0.48 | 0.49 | 0.49 | 1.00 |  |
| Q7 | 0.45 | 0.56 | 0.53 | 0.60 | 0.69 | 0.56 | 1.00 |
| *Residential care* | | | | | | | |
|  | Q1 | Q2 | Q3 | Q4 | Q5 | Q6 | Q7 |
| Q1 | 1.00 |  |  |  |  |  |  |
| Q2 | 0.65 | 1.00 |  |  |  |  |  |
| Q3 | 0.62 | 0.64 | 1.00 |  |  |  |  |
| Q4 | 0.64 | 0.72 | 0.71 | 1.00 |  |  |  |
| Q5 | 0.74 | 0.77 | 0.66 | 0.74 | 1.00 |  |  |
| Q6 | 0.62 | 0.71 | 0.55 | 0.67 | 0.73 | 1.00 |  |
| Q7 | 0.61 | 0.61 | 0.64 | 0.63 | 0.69 | 0.69 | 1.00 |
| *Kinship care* | | | | | | | |
|  | Q1 | Q2 | Q3 | Q4 | Q5 | Q6 | Q7 |
| Q1 | 1.00 |  |  |  |  |  |  |
| Q2 | 0.42 | 1.00 |  |  |  |  |  |
| Q3 | 0.29 | 0.49 | 1.00 |  |  |  |  |
| Q4 | 0.31 | 0.49 | 0.51 | 1.00 |  |  |  |
| Q5 | 0.37 | 0.52 | 0.55 | 0.62 | 1.00 |  |  |
| Q6 | 0.31 | 0.47 | 0.46 | 0.49 | 0.50 | 1.00 |  |
| Q7 | 0.32 | 0.42 | 0.49 | 0.51 | 0.57 | 0.47 | 1.00 |

Online Table 2. Results of Confirmatory Factor Analysis (*N* = 2,795)

| ***Factor loadings*** | **Unstandardised** | **Standardised** |
| --- | --- | --- |
| *Item 1* “I’ve been feeling optimistic about the future” | 1.000 | 0.548^a^ |
| *Item 2* “I’ve been feeling useful” | 1.283 | 0.703 |
| *Item 3* “I’ve been feeling relaxed” | 1.280 | 0.701 |
| *Item 4* “I’ve been dealing with problems well” | 1.357 | 0.743 |
| *Item 5* “I’ve been thinking clearly” | 1.487 | 0.814 |
| *Item 6* “I’ve been feeling close to other people” | 1.200 | 0.657 |
| *Item 7* “I’ve been able to make up my own mind about things” | 1.311 | 0.718 |

Notes: All coefficients significant at p < .001. ^a^Factor fixed at 1.00, unstandardized.

* WEMWBS is protected by copyright. Those wishing to use WEMWBS can obtain a licence to do so. Please go to  [https://warwick.ac.uk/wemwbs/using](https://eur03.safelinks.protection.outlook.com/?url=https%3A%2F%2Fwarwick.ac.uk%2Fwemwbs%2Fusing&data=04%7C01%7CAnthonyRE%40cardiff.ac.uk%7Cf8fad2f17e4a4176c10008d919dff276%7Cbdb74b3095684856bdbf06759778fcbc%7C1%7C0%7C637569272960463357%7CUnknown%7CTWFpbGZsb3d8eyJWIjoiMC4wLjAwMDAiLCJQIjoiV2luMzIiLCJBTiI6Ik1haWwiLCJXVCI6Mn0%3D%7C1000&sdata=%2FzmqJjcmTXw4YnmUHd3BggKygjIEMmJsSk6J9X9crSg%3D&reserved=0) for information on the type of licence you will require and details on how to apply.
